# Identifying Patients With Rapid Progression From Hormone-Sensitive to Castration-Resistant Prostate Cancer: A Retrospective Study

**DOI:** 10.1016/j.mcpro.2023.100613

**Published:** 2023-06-30

**Authors:** Chenxi Pan, Yi He, He Wang, Yang Yu, Lu Li, Lingling Huang, Mengge Lyu, Weigang Ge, Bo Yang, Yaoting Sun, Tiannan Guo, Zhiyu Liu

**Affiliations:** 1 Department of Urology, The Second Hospital of Dalian Medical University, Dalian, China; 2 Center for Intelligent Proteomics, Westlake Laboratory of Life Sciences and Biomedicine, Key Laboratory of Structural Biology of Zhejiang Province, School of Life Sciences, Westlake University, Hangzhou, China; 3 Institute of Basic Medical Sciences, Westlake Institute for Advanced Study, Hangzhou, China; 4 Research Center for Industries of the Future, Westlake University, Hangzhou, China; 5 College of Pharmaceutical Sciences, Zhejiang University, Hangzhou, China; 6 Westlake Omics (Hangzhou) Biotechnology Co., Ltd, Hangzhou, China

**Keywords:** prostate cancer, cancer progression, proteomics, pressure cycling technology, mass spectrometry, data-independent acquisition, machine learning, prognostic analysis

## Abstract

Prostate cancer (PCa) is the second most prevalent malignancy and the fifth cause of cancer-related deaths in men. A crucial challenge is identifying the population at risk of rapid progression from hormone-sensitive prostate cancer (HSPC) to lethal castration-resistant prostate cancer (CRPC). We collected 78 HSPC biopsies and measured their proteomes using pressure cycling technology and a pulsed data-independent acquisition pipeline. We quantified 7355 proteins using these HSPC biopsies. A total of 251 proteins showed differential expression between patients with a long- or short-term progression to CRPC. Using a random forest model, we identified seven proteins that significantly discriminated long- from short-term progression patients, which were used to classify PCa patients with an area under the curve of 0.873. Next, one clinical feature (Gleason sum) and two proteins (BGN and MAPK11) were found to be significantly associated with rapid disease progression. A nomogram model using these three features was generated for stratifying patients into groups with significant progression differences (*p*-value = $1.3\times 10^{-4}$). To conclude, we identified proteins associated with a fast progression to CRPC and an unfavorable prognosis. Based on these proteins, our machine learning and nomogram models stratified HSPC into high- and low-risk groups and predicted their prognoses. These models may aid clinicians in predicting the progression of patients, guiding individualized clinical management and decisions.

## Highlights

• Seventy-eight HSPC patients' proteome with follow-up data on the progression to CRPC.
• Two hundred fifty-one proteins showed differential expression between long- and short-term progressions.
• A 7-protein random forest model can discriminate long- from short-term cases.
• A nomogram model was used for predicting the progression term to CRPC.

## Graphical Abstract

**Figure:**
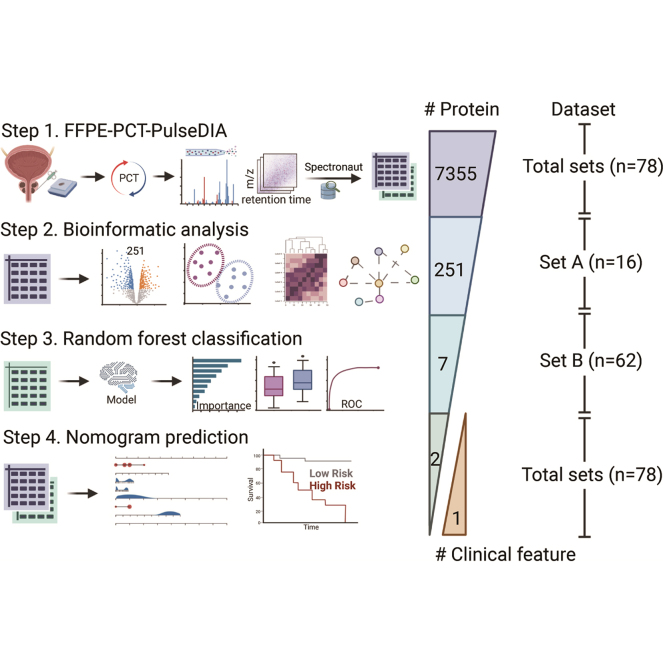

## In Brief

A crucial challenge is to identify the population at risk of rapid progression from hormone-sensitive prostate cancer (HSPC) to lethal castration-resistant prostate cancer (CRPC). Here, we quantified 7355 proteins from 78 patients and identified 251 differential proteins associated with a fast progression to CRPC. Next, we established a machine learning classifier using seven proteins and a nomogram model containing two proteins and a clinical feature to identify patients with rapid progression and predict their prognoses. These models may guide individualized clinical management and decisions.

Prostate cancer (PCa) is the second most prevalent malignancy in males and the fifth leading cause of cancer-related death globally (1). With the implementation of prostate-specific antigen screening (PSA) and the aggravation of population aging, after 2012, the PCa incidence and cancer-related mortality in China began to climb (2). Regarding the risk categories of PCa, locally advanced PCa and metastatic PCa have significantly higher 10- and 15-years mortality rates than other categories (3). Androgen deprivation therapy (ADT) combined with androgen blocking is frequently beneficial to patients with locally advanced and metastatic PCa during the initial treatment stage (4). However, almost all hormone-sensitive prostate cancers (HSPC) progress to castration-resistant prostate cancers (CRPC) within 5 years, with only 5 to 10% of patients remaining alive 10 years after initiating ADT (5).

Due to its heterogeneity, PCa has a complex disease spectrum, ranging from clinically indolent subtypes to aggressive ones. The progress span to CRPC varies significantly among patients; however, limited research has been conducted to explore this. Multiple randomized controlled phase-III trials, including CHAARTED (6) and LATITUDE (7), demonstrated that when patients with HSPC are found to have either a long- or short-term progression to CRPC before initiating treatment, it is possible to implement an early and appropriate follow-up strategy, thereby optimizing treatment regimens. Therefore, it is urgent to accurately predict and identify whether patients with HSPC will progress to CRPC in short term.

The ability to understand the genetics behind large next-generation sequencing datasets has greatly improved (8). However, not all the genetic or transcriptomic aberrations of PCa are translated into the proteome. Specifically, Latonen *et al.* (9) reported that gene copy numbers, DNA methylations, and RNA expression levels do not reliably predict the proteomic changes of PCa, especially CRPC. By quantifying and validating large numbers of proteins, their findings indicate that proteomics could be a more promising approach for identifying the molecular mechanisms underlying HSPC progression to CRPC.

In recent years, pressure cycling technology (PCT) and pulsed data-independent acquisition (PulseDIA) have enabled the in-depth and fast identification of proteomes in trace amounts of clinical biopsies, not only fresh frozen (10) but also formalin-fixed and paraffin-embedded (FFPE) ones (11). Through the use of FFPE clinical biopsies tissue, PCT-PulseDIA tackles the problem that the data collecting period is too long owing to the collection of clinical prognosis information, therefore considerably speeding up the understanding of major diseases or crucial phases of clinical research.

This study explored the proteomic differences between HSPC patients who experience either a long- or short-term progression to CRPC. To this aim, we used the PCT-PulseDIA pipeline to analyze the proteomes of pre-ADT PCa biopsies. In addition, the proteomes of HSPC patients with significantly distinct progression were retrospectively evaluated along with clinical data. Finally, we generated a machine learning model for classifying patients as undergoing a long- or short-term progression to CRPC and a nomogram model for predicting the progression risk of HSPC patients.

## Experimental Procedures

### Ethics Statement

A total of 78 HSPC patients with complete follow-up information were recruited between January 2014 and July 2021 at the Second Hospital of Dalian Medical University. The clinical characteristics of these patients are given in supplemental Table S1. This research was approved by the ethical committee of the Second Hospital of Dalian Medical University with the Declaration of Helsinki. The study was registered in the Chinese Clinical Trial Register (ChiCTR2100054836), and all patients signed a written informed consent before participation.

### Patient Diagnosis, Treatment and Follow-Up

Locally advanced PCa patients were classified according to the European Association of Urology Prostate Cancer Guideline as any PSA, cT3-4 or cN+, any International Society of Urological Pathology (ISUP) grade or Gleason sum (GS), while metastatic PCa was defined as cM1 disease based on CT and bone scans (12, 13). Accordingly, the patients from our study population were diagnosed with locally advanced (N = 4) or metastatic disease (N = 74) based on histopathological biopsies.

Subsequently, all patients were treated with ADT (luteinizing hormone-releasing hormone agonists: goserelin 3.6 mg, once every 28 days, one dose each time, or leuprorelin 3.75 mg, once every 28 days, one dose each time, subcutaneous injection in numerous areas of the upper arm, belly, and buttocks) and with anti-androgen (bicalutamide 50 mg s.i.d. or flutamide 250 mg t.i.d., taken orally). This was the initial and only therapy before progression. Those patients that progressed to CPRC met the following criteria: (1) serum testosterone level <50 ng/dl, or 1.7 nmol/L; (2) PSA progression: monitoring serum PSA levels at least three times consecutively at one-week intervals, with the serum PSA continuously increasing and each subsequent reading showing an increase of over 50% compared to the baseline value and the absolute value of PSA reaching above 2.0 ng/ml. All patients were followed up till their advancement to CRPC. Patients with cardiovascular diseases, autoimmune diseases, other malignancies, or deceased due to other causes were excluded. The GS was used to evaluate each PCa case's malignancy and annotate primary and secondary patterns.

### Experimental Design and Statistical Rationale

**Fig. 1:**
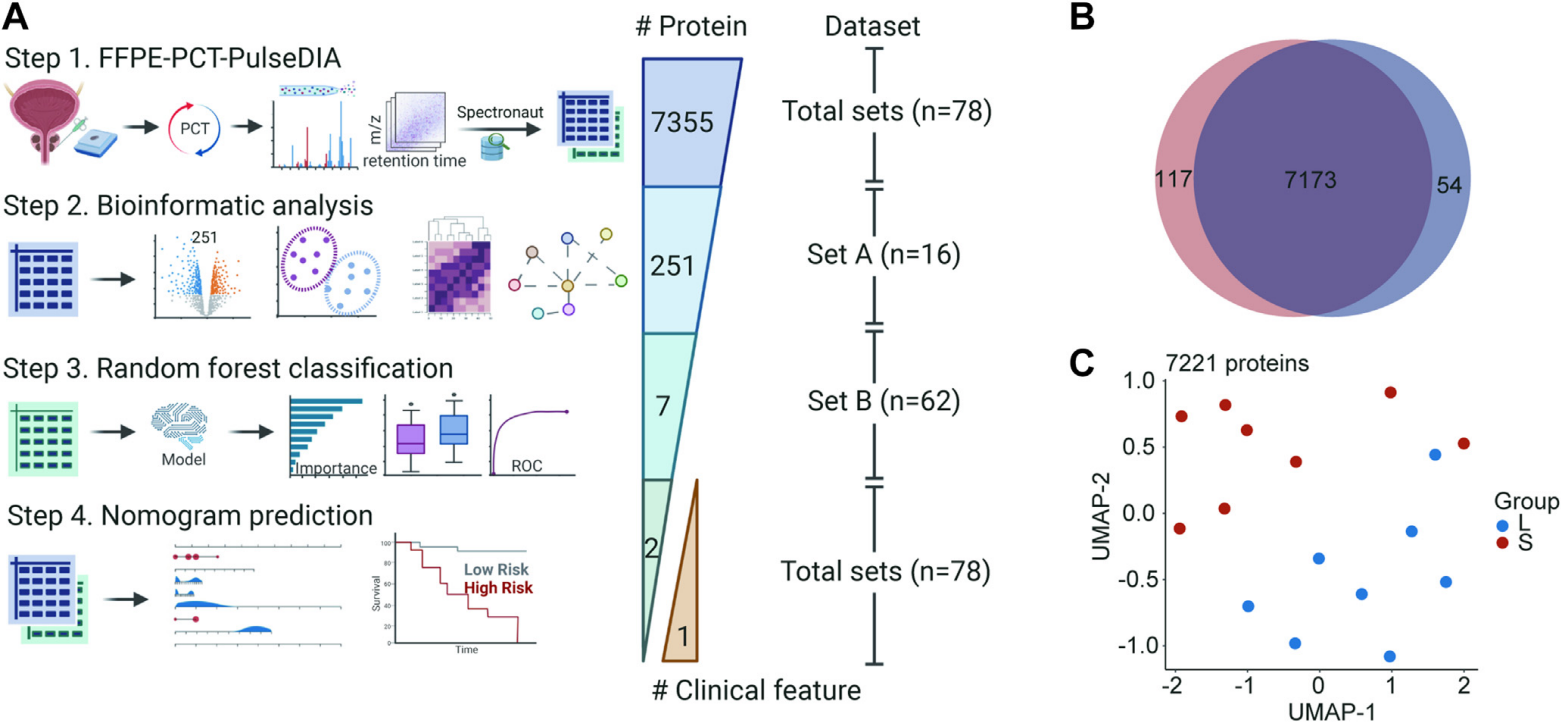
**Study design and global proteomics view of thesetA.***A*, workflow of the study. Created from Biorender.com*B*, venn diagram of the protein identifications from the short- (S) and long-term (L) progression groups. *C*, UMAP plot showing the sample distribution of the S and L groups based on 7221 proteins with less than 85% missing values.

This study included four phases (Fig. 1*A*): (1) FFPE-PCT-PulseDIA, (2) Bioinformatic analyses, (3) Random forest classification, and (4) Nomogram prediction.

For the ﬁrst phase, the pathologists evaluated the primary Gleason patterns and detailed the boundaries of the cancerous changes on the FFPE slides. The pre-ADT samples were punched (diameter 1 mm) from the area of interest of FFPE blocks, thus ensuring that all samples were derived from cancerous tissues. A total of 78 tissue cores from 78 patients with HSPC were collected and then assigned to two groups: a set A (n = 16) and a set B (n = 62). These samples were then processed by PCT-PulseDIA workflow. For data quality control, we acquired and analyzed four technical replicates from the same pooled peptide sample to confirm the stability of the mass spectrometer.

Regarding the second phase, to generate a model for stratifying the patients with long- or short-term progression, we screened the differentially expressed proteins (DEPs) from the set A with *p*-value < 0.01 and fold-change > 2, based on the set A which including long- (L, n = 8) and short-term (S, n = 8) progression cases. To generate a machine learning model and a nomogram, the resulting 251 DEPs were preserved in the set B, and their missing values and batch effects were processed by SeqKNN and ComBat algorithms.

In the third phase, the set B was separated into a training set of 41 samples (∼2/3), and an independent testing set of 21 samples (∼1/3). According to the median progression time (9 months) from HSPC to CRPC, the label of these samples was further divided into group S and group L. We conducted an analysis of variance (ANOVA) on the training set for each DEP, selected the significant proteins whose corresponding *p*-values were less than 0.05 according to the ANOVA, constructed a random forest (RF) model using the seven significant proteins based on the training set, and validated the RF model using the independent testing set. Specifically, we created 500 trees, set the node size hyperparameter to one, selected the Gini index as the importance metric for the variables, and left all the other hyperparameters at their default values. We used “mlr3” R package for the above machine learning analysis.

In the last phase, the multivariable Cox regression (R package "survival") was used to determine the prognostic significance of 13 features, comprising six clinical characteristics (age, T-stage, N-stage, M-stage, total PSA (tPSA), GS) and seven proteins from the RF model: Mitogen-Activated Protein Kinase 11 (MAPK11), CDGSH Iron Sulfur Domain 1 (CISD1), Wolframin ER Transmembrane Glycoprotein (WFS1), Transcription Elongation Factor A Like 1 (TCEAL1), Biglycan (BGN), Zinc Finger Protein 70 (ZNF70), and RNA Polymerase III Subunit B (POLR3B). In addition, the R package "forestplot" was used to visualize each variable (including *p*-value, hazard ratio (HR), and 95% confidence interval (CI)). Next, the nomographs were created using the "RMS" software to predict the disease progression rates after 12, 18, and 24 months. The calibration curve showed the performance of the nomograms with the observed rates at 12, 18, and 24 months. The optimal cutoff value of the risk score was calculated using the R package "maxstat". We set the minimum number of samples in each group to be greater than 25% and the maximum number of samples in each group to be less than 75%. Patients were further classified into high or low-risk groups based on this criterion with a cutoff of 0.44. The prognostic difference between the two groups was estimated using the "Survfit" function in the "survival" package and the log-rank test. Finally, we obtained the area under the curve (AUC) by the receiver operating characteristic (ROC) analysis using the R package "pROC". In particular, we gathered the patients’ follow-up durations and risk scores, and performed the ROC analyses at 12, 18, and 24 months.

### Proteomics Sample Preparation

The PCT-assisted proteomics sample preparation procedures followed our previously published workflows (14). In brief, about 0.2 mg of FFPE punches were dewaxed with heptane, hydrated with ethanol, and then underwent acidic hydrolysis by 0.1% formic acid (FA, Thermo Fisher Scientific) and basic hydrolysis by 0.1 M Tris-HCl (pH = 10.0). Samples were next lysed using a 6 M urea/2 M thiourea buffer (Sigma), reduced by tris (2 carboxyethyl) phosphine (Sigma), and alkylated by iodoacetamide (Sigma). The lysates were then digested using PCT by a mix of Lys-C and trypsin (Hualishi Tech. Ltd). Finally, the PCT-assisted digestion reaction was stopped by trifluoroacetic acid and cleaned by C18.

### HSPC-Specific Spectral Library Establishment

To analyze the PulseDIA data, we generated an experimental spectral library for the PCa tissues. We combined the cleaned peptides from the set A into a mixture containing 100 μg peptides. The peptide pool was then separated using Thermo Ultimate Dinex 3000 (Thermo Fisher Scientific) with an XBridge Peptide BEH C18 column (300 Å, 5 μm × 4.6 mm × 250 mm; Waters) and a 60-min gradient. Finally, we collected 20 peptide fractions. The fraction data were acquired using data-dependent acquisition (DDA).

Spectronaut with Pulsar engine (software version 14.6, engine version 13.18.44, Biognosys) was used to generate the HSPC-specific spectral library (15). The DDA files were searched by Pulsar against a human Swiss-Prot FASTA database (downloaded on 2020-01-22), including 20,367 protein sequences with a false discovery rate (FDR) of 0.01 at PSM, peptide, and protein levels. Specific enzyme cleavage “trypsin/P; LysC” was considered, allowing for no more than two missed cleavages; “cysteine carbamidomethyl” was set to a fixed modification, while “methionine oxidation” and “protein N-terminal acetylation” were set to variable modifications; mass tolerance was automatically determined; and the remaining settings were left to their default values.

### PulseDIA Data Acquisition and Data Processing

A total of 400 ng peptides were injected and separated along a 45 min liquid chromatography gradient (from 3 to 28% buffer B – see below for its composition) at a flow rate of 300 nl/min (precolumn: 3 μm, 100 Å, 20 mm × 75 μm i.d.; analytical column: 1.9 μm, 120 Å, 150 mm × 75 μm i.d.). Buffer A was mass spectrometry-grade water containing 2% acetonitrile and 0.1% FA; buffer B was acetonitrile containing 2% water and 0.1% FA. The peptides were then analyzed by a Q Exactive HF hybrid Quadrupole-Orbitrap (Thermo Fisher Scientific) using the PulseDIA mode with four pulses, as previously described (16). MS1 was performed over an *m/z* range of 390 to 1210 Th with a resolution of 60,000 FWHM, an automatic gain control (AGC) target of 3E6, and a max ion injection time (IT) of 80 ms. The PulseDIA isolation windows were complementary and discontinuous for the same set of pulse injections and contained 1 *m/z* overlap between adjacent windows. MS2 was set with a resolution of 30,000 FWHM, an AGC target of 1E6, and a max IT of 50 ms.

PulseDIA analysis was performed on Spectronaut (version 14.6) according to the standard workflow from Spectronaut manual. Specifically, the MS1 and MS2 mass tolerance were set as dynamic. The local (nonlinear) regression was set to correct retention time prediction. Spectronaut will determine the ideal extraction window dynamically depending on iRT calibration and gradient stability. Sections of the gradient that show higher variability during the calibration step will automatically be extracted using wider windows. At least three fragment ions were calculated for peptide identification and major and minor group quantities were set to mean peptide and mean precursor quantity. The FDR was set to 1% at the peptide and protein level, while other settings were default.

### Statistical and Bioinformatic Analysis

Before our data analysis, the protein matrix was log_2_-transformed. The coefficients of variation (CVs) of the proteins across the pooled samples and the Spearman correlation coefficients between pairs of pooled samples were then used to evaluate the data quality. Specifically, missing values were excluded when calculating the CVs and correlations. The *t* test was used for selecting the DEPs. Pathway and gene ontology (GO) enrichment were performed using Ingenuity Pathways Analysis (IPA, database version of Spring Release, 2023) and Cytoscape (version 3.9.1) with the ClueGO plugin (version 2.5.9).

## Results

### Study Design and Global View of Our Proteomics Analysis

The whole study was divided into four stages, as described in the Experimental Design and Statistical Rationale section (Fig. 1*A*). To stratify the patients with long- or short-term progression from HSPC to CRPC, in the first stage, we collected and analyzed two sets of pre-ADT PCa samples (n = 78): a set A (n = 16) and a set B (n = 62). From the entire data sets (set A and set B), we identified 93,440 peptides, 7355 protein groups, and 7306 proteotypic proteins (excluding single peptide support proteins).

Next, we compared the proteomic expression differences between the two main groups in the set A: the long- (Group L, n = 8) and short-term (Group S, n = 8) progression groups. Specifically, Group L had shown a progression of at least 20 months, while Group S had a progression of no more than 8 months. This time refers to the interval between the diagnosis of PCa and the diagnosis of CRPC. A total of 81,482 peptides, 7344 protein groups, and 7295 proteotypic proteins were identified in the 16 samples. An average of 43,930 peptides and 6114 protein groups were detected. In particular, the median numbers of peptides and protein groups were 39,514 and 6026 for Group L and 52,081 and 6624 for Group S (supplemental Fig. S1, *A* and *B*). A total of 97.7% (7173/7344) proteins were identified across both groups. In contrast, 117 and 54 proteins were exclusively expressed in Group S and Group L, respectively (Fig. 1*B*). The higher number of unique proteins identified in Group S may be explained by the more severe condition of the disease.

Next, we kept the proteins with less than 85% missing values, i.e., that were detected in at least three samples, resulting in 7221 proteins with relatively high confidence. The two sample groups were partially separated in the global view of the UMAP plot of the 7221 protein features (Fig. 1*C*). This result suggests biological differences between the two groups.

### Proteomic Characteristics and Differences Between Patients With Long- and Short-Term Progression

**Fig. 2:**
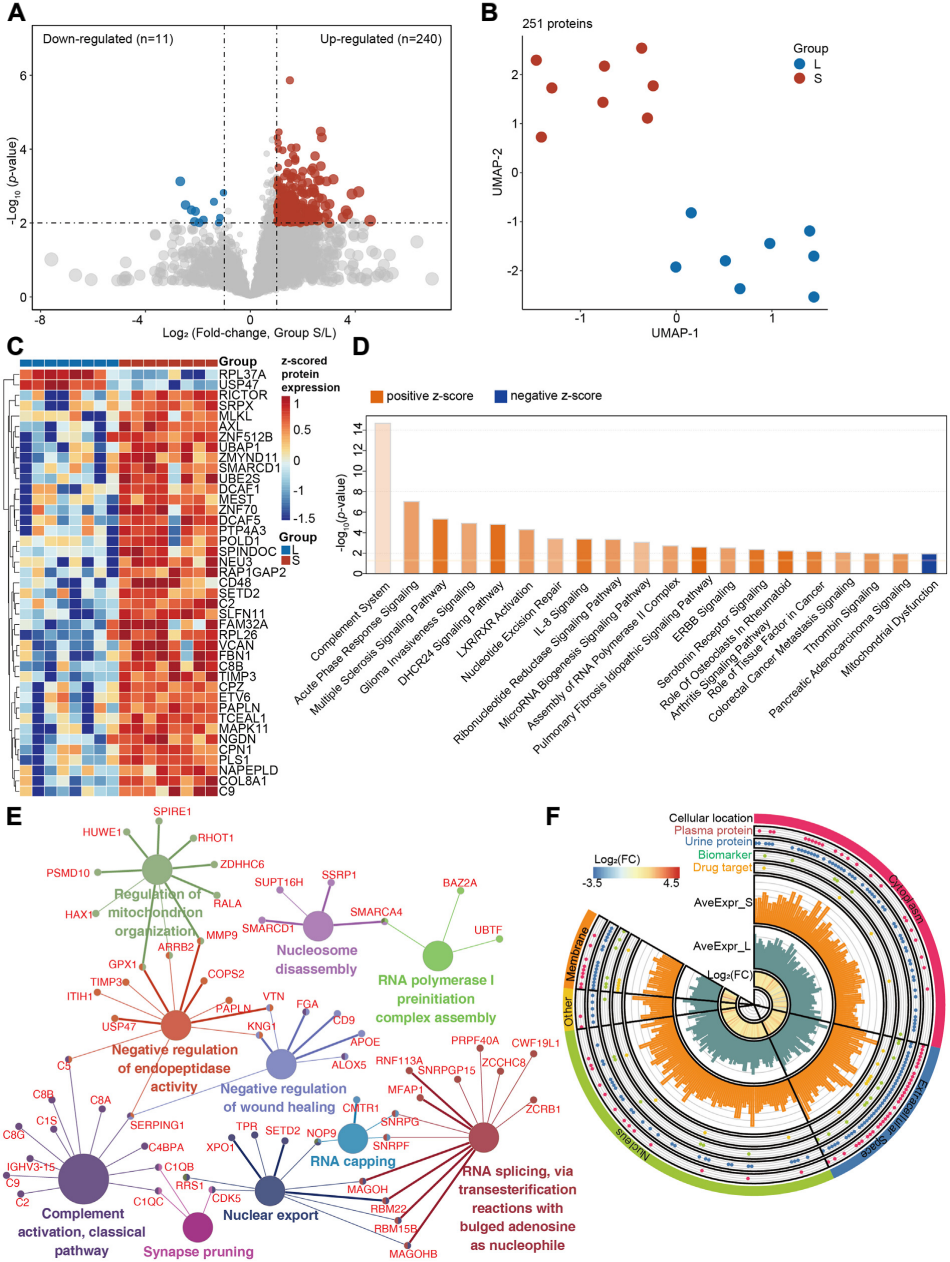
**Comparative proteomics and bioinformatic analysis of thesetA.***A*, volcano plot of the 251 differentially expressed proteins (DEPs) between Group S and Group L with *p*-value < 0.05 and fold-change >2. *B*, using the 251 DEPs, the two groups were separated in the UMAP plot. *C*, heatmap showing the expression of the 41 most significantly dysregulated proteins for each sample. *D*, pathway enrichment of the 251 DEPs. Positive and negative z-scores indicate the active and inhibited pathways, respectively. *E*, the gene ontology enrichment networks of the 251 DEPs generated by the ClueGO plugin of Cytoscape. *F*, circos plot showing the 251 DEPs annotated by cellular location, their being plasma proteins, urine proteins, biomarkers, and/or drug targets, their average expression in Group S (AveExpr_S) and Group L (AveExpr_L), and their log_2_(fold change, FC).

To explore the biological difference between the long- and short-term progression patients, we first identified the dysregulated proteins and visualized them using a volcano plot (Fig. 2*A*). This comparison revealed 11 downregulated and 240 upregulated proteins in Group S with fold-changes >2 and *p*-values < 0.01 (Fig. 2*A*). The two groups could be clearly distinguished in the UMAP plot based on these 251 DEPs (Fig. 2*B*). The heatmap in Figure 2*C* shows the expressions of the 41 most significantly dysregulated proteins.

Next, the 251 DEPs were enriched using pathway and GO analyses. The signaling pathways of the complement system, the acute phase response, and the multiple sclerosis signaling pathways were the three most significantly activated in Group S. Also, the pathways involved in mitochondrial dysregulation signaling were inhibited in Group S (Fig. 2*D*). Our GO enrichment analysis showed that, besides the complement activation, several other processes are also involved in an unfavorable prognosis of the disease: regulation of mitochondrion organization, synapse pruning, negative regulation of endopeptidase activity, negative regulation of wound healing, nuclear export, nucleosome disassembly, RNA capping, RNA splicing, and RNA polymerase I preinitiation complex assembly (Fig. 2*E*). The above data revealed the most important signaling pathways, biological processes, and the corresponding proteins that are associated with a rapid disease progression. The dysregulated proteins we identified have been reported in multiple biofluids, and they could potentially be used as diagnosis or prognosis biomarkers and therapeutic drug targets (Fig. 2*F*).

### Identifying Patients With Rapid Progression Using a Protein Panel-Based Machine Learning Model

To stratify the two groups using statistical models, we collected a larger sample set: the set B (n = 62). Using these samples and the same analytical methods used for the set A, we identified 7353 proteins; the quality control analysis proved the data quality to be satisfactory (median CV = 0.0377; correlation coefficients >0.85) (supplemental Fig. S1, *C* and *D*).

**Fig. 3:**
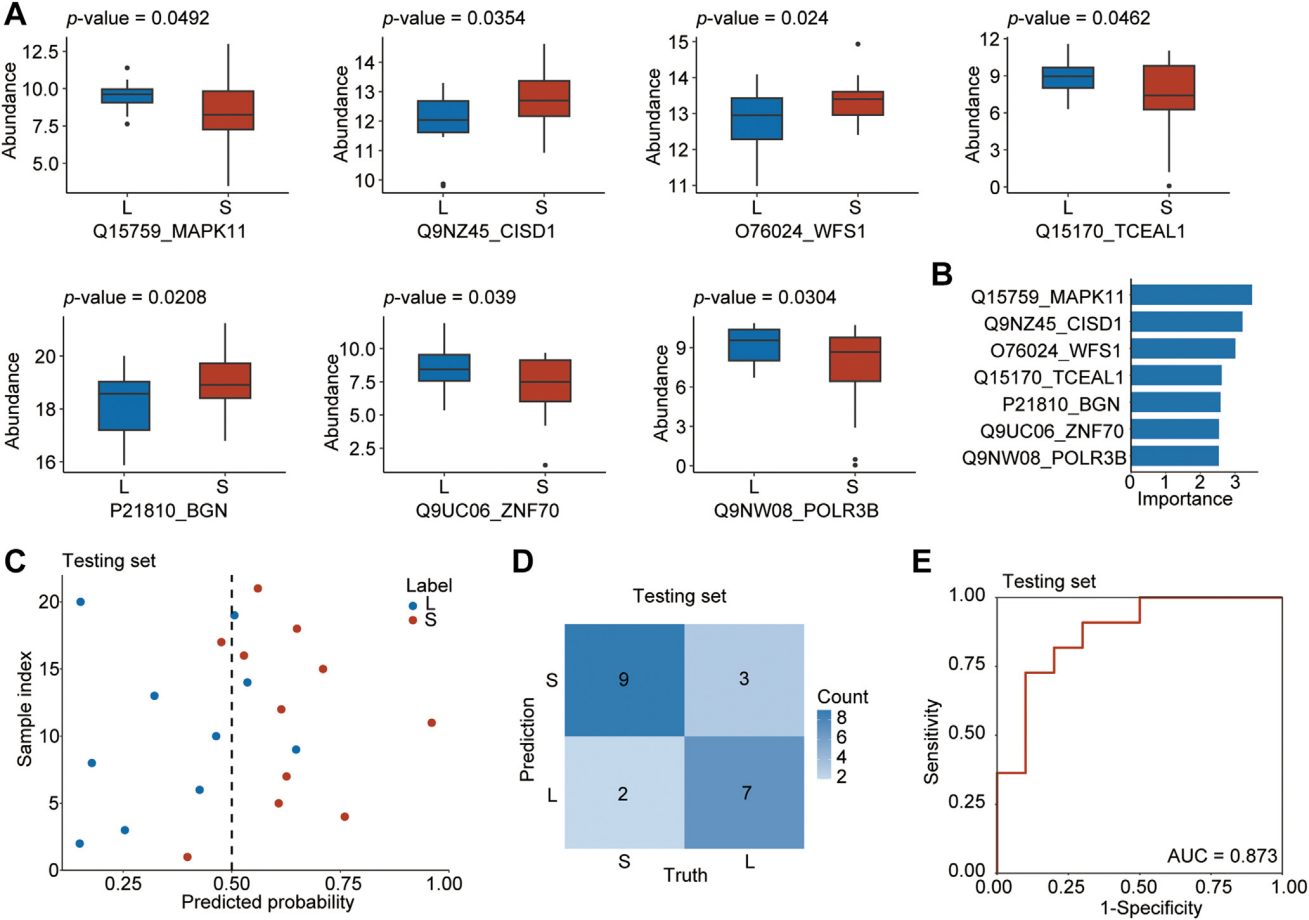
**Stratification and prediction for patients with short- and long-term progression.***A*, boxplots showing the log_2_ (protein abundance) in Groups S and L. The *p*-values were calculated using ANOVA. *B*, the gini importance of each protein feature ranked by our random forest model. *C*, the predicted probabilities of belonging to Groups S for the patient from our testing set. Our random forest model generated these results. *D*, confusion matrix of the independent testing set. *E*, ROC plot showing the model performance on an independent testing set.

Within the training set (n = 41), we identified the most significant proteins by setting the ANOVA *p*-values < 0.05 and then selected seven proteins with statistically significant differences. Among these, four proteins were significantly downregulated, and the other three were significantly upregulated in Group S (*p*-value < 0.05) (Fig. 3*A*). We then built our RF model and ranked these seven protein features based on their Gini importance in our model (Fig. 3*B*). The model performance on the training set was shown in the supplemental Fig. S2. Next, each individual in the testing set (n = 21) was scored by the RF model based on the above seven features (Fig. 3*C*). The resulting model correctly classified 16 of 21 patients (testing set) with an accuracy of 0.762 (Fig. 3*D*); the model's sensitivity, specificity, positive predictive value, and negative predictive value were 0.818, 0.700, 0.750, and 0.778, respectively. The AUC of our RF model was 0.873 (Fig. 3*E*), showing the high performance of our classification model.

### Predicting the Progression Time and Risk Through Nomogram

**Fig. 4:**
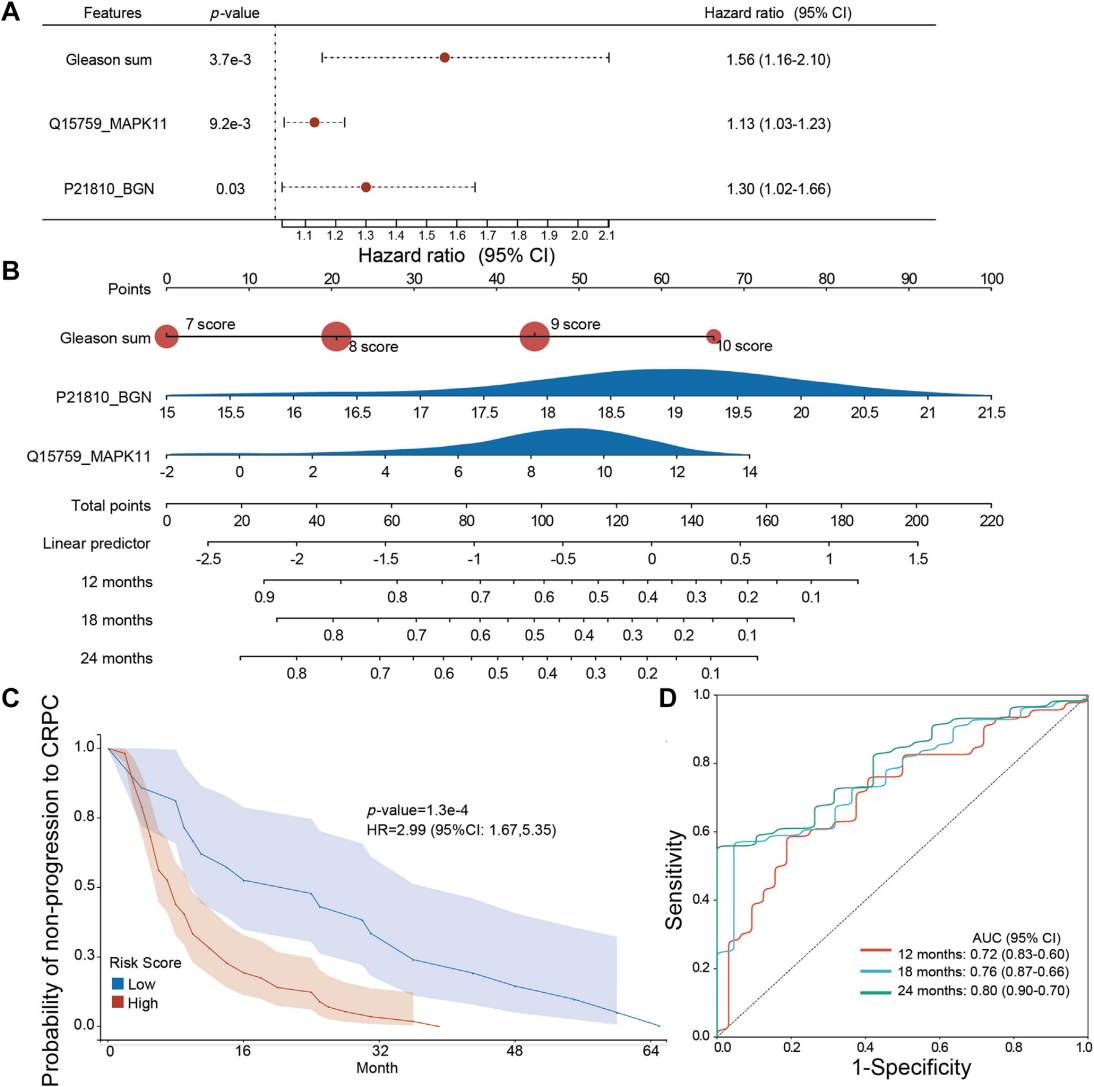
**Predicting the probability of non-progression to castration-resistant prostate cancer (CRPC) at different times.***A*, multivariable Cox analysis of the proteins and clinical features selected for distinguishing progression to CRPC (*p*-value < 0.05). *B*, nomogram for the prognostic prediction of developing advanced hormone-sensitive prostate cancer. *C*, survival curves indicating the probability of non-progression to CRPC. The low- and high-risk are grouped using an optimal nomogram risk score cutoff of 0.44. *D*, ROC curves for the 12-, 18-, and 24-month prostate cancer progress-related nomogram.

We next predicted the progression time and the risk of progression to CRPC for each individual. To this aim, we screened the clinical characteristics and the above seven protein features using a multivariable Cox proportional hazards regression. Three of the 13 features were significantly associated with an unfavorable prognosis. Specifically, one clinical feature (GS) and two protein features (BGN and MAPK11) were positively associated with the disease progression. (Fig. 4*A*). Hence, these three features may help predict the probability of individual PCa cases rapidly progressing into CRPC.

Next, we constructed a nomogram by integrating these three features and predicted the disease progression (Fig. 4*B*). Each individual was scored using our nomogram and the three features, with a risk score cutoff of 0.44. Specifically, based on this cutoff, patients were split into groups of high- (n = 21, ratio of 27%) and low-risk scores (n = 57, ratio of 73%). The model's overall concordance index (C-index) was 0.67 (95% CI 0.60–0.74), and our calibration plot showed the agreement between our estimations and the observations 18 and 24 months after being confirmed as HSPC (supplemental Fig. S3). The progression curve showed a statistically significant difference between the two groups (*p*-value = $1.3\times 10^{-4}$, HR = 2.99) (Fig. 4*C*). The AUC values of the 18-month (AUC = 0.76) and 24-month (AUC = 0.80) nomogram progressions were larger than that of the 12-month (AUC = 0.72) one (Fig. 4*D*). Therefore, the most accurate nomograms for predicting the development of PCa were the 18-month and 24-month nomograms.

## Discussion

In this study, we investigated patients with pre-ADT PCa by exploring the differences between long- and short-term progression cases. We collected clinical data and measured the proteomes of PCa biopsies. We newly identified proteins that may be crucial in a faster progression of HSPC to CRPC. Finally, we generated a model for predicting the advancement of HSPC and stratifying patients into high- and low-risk groups.

The clinical staging of newly diagnosed patients with PCa in China differs from Western developed countries. For instance, among the newly diagnosed patients with PCa in the United States, most cases (∼76%) are clinically localized, while only ∼13% and ∼6% involve metastases in local lymph nodes or distant sites, respectively (17). However, the data in China are quite different. A multicenter Chinese study showed that only one-third of the newly diagnosed patients with PCa are clinically localized. Also, most patients in China are in the middle or advanced stage at the diagnosis of PCa, resulting in a worse overall prognosis than in Western countries (18). For this reason, our study focused on exploring advanced PCa cases from China. In our study, we enrolled 78 patients with advanced PCa, but their median time for progressing to CRPC was only 9 months: much shorter than previously published (19). This fact may be explained by most of our enrolled patients already having developed metastases (N = 72, 97.3%).

Recent studies have shown that the time of HSPC progression to CRPC is highly variable in patients treated with standard ADT (20). Multiple phase-III trials proved the importance of identifying patients with HSPC at risk of rapid disease progression, allowing for the early implementation of appropriate therapeutic strategies to improve the prognosis (21). There are two well-known studies on the risk classification of metastatic HSPC. The first was the CHAARTED trial, where patients with visceral and/or at least four bone metastases were classified as high-volume to distinguish them from the remaining low-volume ones (6), concluding that the high-volume group benefits from ADT + docetaxel treatment, whereas the low-volume group should be served by ADT alone. The second study was the LATITUDE trial, where patients with at least two high-risk characteristics (at least three bone metastases, visceral metastases, and ISUP grade four) were categorized as high-risk, which were found to have an increased survival when following an abiraterone acetate plus prednisone therapy (7). However, it is obvious that there were many patients defined as low-risk or low-volume, who progressed to CRPC rapidly in the above research. And in our study, most patients would have been classified as low-volume and low-risk according to the CHAARTED and LATITUDE criteria, respectively. However, our patients had a rapid disease progression (median time: 9 months). Collectively, stratifying patients based on clinical imaging or M stage alone may be inappropriate.

In this study, a novel nomogram was established for predicting the probability of fast progression to CRPC, by integrating two proteins (BGN and MAPK11) and one clinical feature (GS), based on their expression levels and regression coefficients, as demonstrated by the multivariate Cox regression analysis. As a scoring system, of which predictive ability was confirmed by the overall C-index and the AUC values, our results suggest a better discrimination capability than previously published transcriptomic signatures consisting of two to 22 genes (22, 23, 24, 25).

According to the risk score, patients were then divided into high-risk and low-risk groups. The Kaplan–Meier survival curve showed that patients with high-risk scores had significantly poorer progression-free survival than those with low-risk scores, suggesting that patients with high-risk scores were more prone to progression. According to the current clinical trials mentioned earlier, high-risk patients may respond better to ADT combined with chemotherapy or novel endocrine therapy medicines. Larger scale clinical trials are necessary to assess the most suitable treatments for each identified category.

In terms of the two proteins in the model, BGN and MAPK11 have been proven to promote the occurrence and progression of prostate cancers, which were consistent with our results. BGN has gained interest because it is part of a commercial RNA expression signature for estimating prostate cancer aggressiveness (26). Some researchers have pointed out that upregulation of biglycan is associated with a poor prognosis and PTEN deletion in prostate cancer (27). In osteolytic prostate cancer cells, MAPK11 correlates with Dickkopf-1 expression in different stages of prostate cancer and is associated with prostate cancer metastasis (28). At the same time, MAPK/p38 inhibitors have been further studied in the treatment of prostate cancer (29).

Although several proteomics studies of PCa have been published, they mainly focused on characterizing protein alterations and their biological changes by comparing benign/normal with PCa cases (30), exploring metastatic PCa (31), describing the heterogeneity of PCa (32), and investigating the disease progression (9). Ours is the first study to employ proteomics to investigate the differences between long- and short-term progressions from HSPC to CRPC and to discover a panel biomarker for identifying patients with a rapid progression. The ∼7500 proteins we identified provide a high-quality resource for explorative analyses. Furthermore, the 251 DEPs we found associated with different progressions to CRPC were enriched in the complement system and other inflammatory response-related pathways and functions (Fig. 2, *D* and *E*), in agreement with previous findings using prostatic fluids (33). Previous studies have confirmed a significant association between complement activation and prostate cancer development or progression (34) and have confirmed that during disease progression from primary PCa to metastatic PCa, especially bone metastases, changes in extracellular matrix composition favor tumor spread and colonization at distant sites (35).

Our study was limited by the small sample size, which was collected from a single center. Also, all our patients were treated with standard ADT. However, in clinical practice, ADT, combined with bicalutamide and flutamide, is gradually being replaced by the combination of chemotherapy and second-generation hormonotherapy. Therefore, further validations need to be performed on a larger and multicenter study. In particular, individuals with different treatment approaches should be compared to determine the benefits of various treatment strategies in connection with patient stratification.

## Conclusion

We identified proteins and clinical parameters that are significantly associated with a fast progression of HSPC to CRPC. Using this information, we developed efficient models for classifying patients with PCa and predicting HSPC development. Our data and models can potentially guide the clinical management of patients with HSPC.

## Data Availability

The mass spectrometry proteomics data have been deposited to the iProX with the dataset identifier IPX0005031000.

## Supplemental data

This article contains supplemental data.

## Conflict of interest

The authors declare the following financial interests/personal relationships which may be considered as potential competing interests: T. G. is a shareholder of Westlake Omics Inc. L. H. and W. G. are employees of Westlake Omics Inc. T. G., Z. L., Y. S., Y. H., H. W., and C. P. have applied for a patent on this project. The remaining authors declare no competing interest.
