# Supplementary material for: Identifying Patients With Rapid Progression From Hormone-Sensitive to Castration-Resistant Prostate Cancer: A Retrospective Study
Source: Mol Cell Proteomics. 2023 Jun 30;22(9):100613. doi: 10.1016/j.mcpro.2023.100613 (PMC10491655; doi:10.1016/j.mcpro.2023.100613)
Supplement: Supplemental Figures S1–S3 [file mmc1.docx]

Supplementary Materials

Identifying patients with rapid progression from hormone-sensitive to castration-resistant prostate cancer: a retrospective study

## Authors

Chenxi Pan^1#^, Yi He^1#^, He Wang^2,3,4#^, Yang Yu^1#^, Lu Li^2,3,4,5^, Lingling Huang^6^, Mengge Lyu^2,3,4^, Weigang Ge^6^, Bo Yang^1^*, Yaoting Sun^2,3,4^*, Tiannan Guo^2,3,4^*, Zhiyu Liu^1^*

## Affiliations

^1^Department of Urology, The Second Hospital of Dalian Medical University, Dalian, China;

^2^Center for Intelligent Proteomics, Westlake Laboratory of Life Sciences and Biomedicine, Key Laboratory of Structural Biology of Zhejiang Province, School of Life Sciences, Westlake University, Hangzhou, China;

^3^Institute of Basic Medical Sciences, Westlake Institute for Advanced Study, Hangzhou, China;

^4^Research Center for Industries of the Future, Westlake University, Hangzhou, China;

^5^College of Pharmaceutical Sciences, Zhejiang University, Hangzhou, China;

^6^Westlake Omics (Hangzhou) Biotechnology Co., Ltd., Hangzhou, China;

^#^Co-first authors;

^*^Co-Corresponding authors;

Correspondence: zyliu@dmu.edu.cn

**Supplemental Figure 1. Data quality control analysis.** (A) Numbers of the identified proteins and peptides from Group S and Group L in the set A. (B) Statistical analysis for the peptide and protein identifications in the set A. (C) Coefficients of variation of the proteins across the pooled samples in the set B. (B) Spearman correlations between pairs of pooled samples in the set B.

**Supplemental Figure 2. Model performance on the training set.** (A) The predicted probabilities of belonging to Groups S for the patient in our training set. Our random forest model generates these results. (B) The confusion matrix of the training set. (C) The receiver operating characteristic curve of the training set.

**Supplemental Figure 3. The calibration curves of our nomogram.** Calibration curves for the 12-, 18-, and 24-month progressions to advanced prostate cancer.
