# Supplementary material for: Identifying Patients With Rapid Progression From Hormone-Sensitive to Castration-Resistant Prostate Cancer: A Retrospective Study
Source: Mol Cell Proteomics. 2023 Jun 30;22(9):100613. doi: 10.1016/j.mcpro.2023.100613 (PMC10491655; doi:10.1016/j.mcpro.2023.100613)
Supplement: Supplemental Table S1 [file mmc2.docx]

**Supplemental Table 1. Clinical characteristics of study participants**

|  | **Clinical characteristics** | **Number** |
| --- | --- | --- |
| **Age** | Mean±SD | 72.90±8.68 |
|  | Median [min-max] | 74.00 [50.00,89.00] |
| **Gleason** | 5+5 | 4 (5.13%) |
|  | 5+4 | 16 (20.51%) |
|  | 5+3 | 3 (3.85%) |
|  | 4+5 | 12 (15.38%) |
|  | 4+4 | 20 (25.64%) |
|  | 4+3 | 14 (17.95%) |
|  | 3+5 | 5 (6.41%) |
|  | 3+4 | 4 (5.13%) |
| **T-stage** | T4 | 40 (51.28%) |
|  | T3 | 25 (32.05%) |
|  | T2 | 13 (16.67%) |
| **N-stage** | N1 | 53 (67.95%) |
|  | N0 | 25 (32.05%) |
| **M-stage** | M1c | 9 (11.54%) |
|  | M1a/b | 61 (78.20%) |
|  | M0 | 8 (10.26%) |
| **Time to CRPC (months)** | Mean±SD | 14.97±14.12 |
|  | Median [min-max] | 9.00 [2.00,65.00] |
| **Gleason sum** | 9 | 28 (35.90%) |
|  | 8 | 28 (35.90%) |
|  | 7 | 18 (23.08%) |
|  | 10 | 4 (5.13%) |
| **t-PSA** | < 50 | 11 (14.10%) |
|  | 50-100 | 15 (19.23%) |
|  | > 100 | 52 (66.67%) |
