# Supplementary figures and images for: Identifying Patients With Rapid Progression From Hormone-Sensitive to Castration-Resistant Prostate Cancer: A Retrospective Study
Source: Mol Cell Proteomics. 2023 Jun 30;22(9):100613. doi: 10.1016/j.mcpro.2023.100613 (PMC10491655; doi:10.1016/j.mcpro.2023.100613)

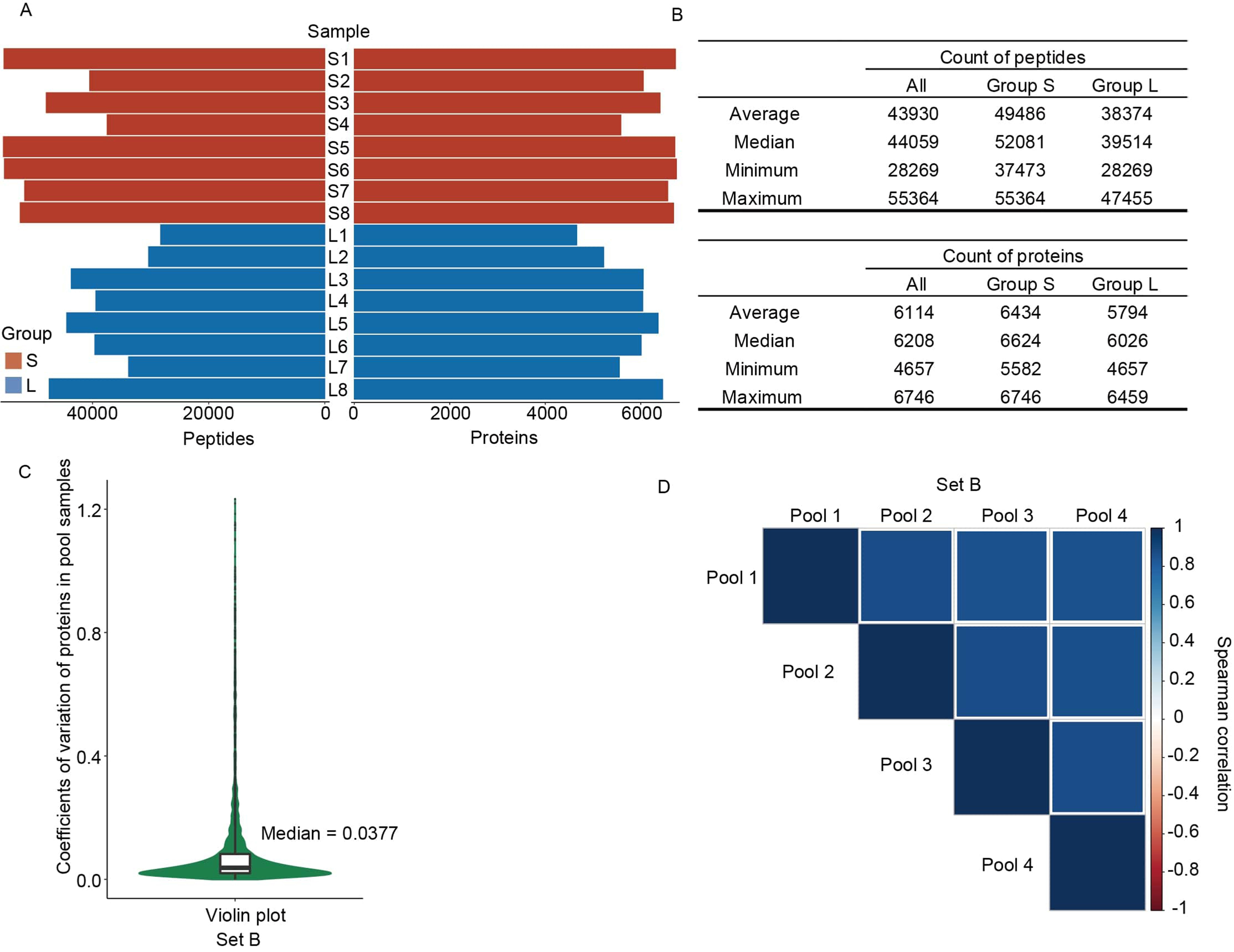

Supplement: Supplemental Figure S1 [file figs1.jpg]

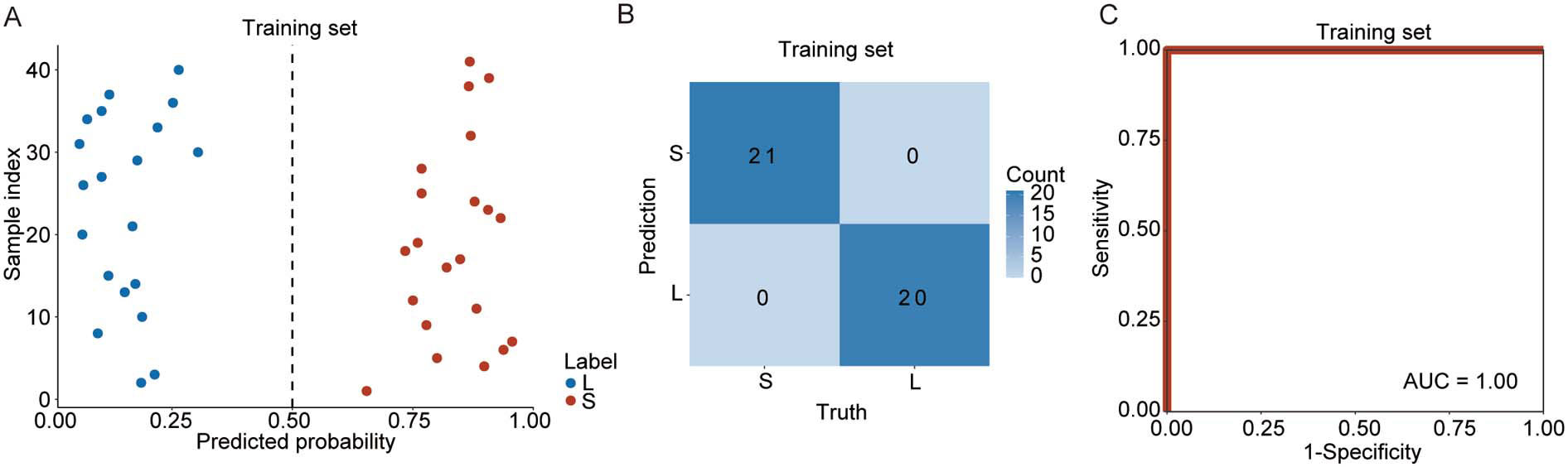

Supplement: Supplemental Figure S2 [file figs2.jpg]

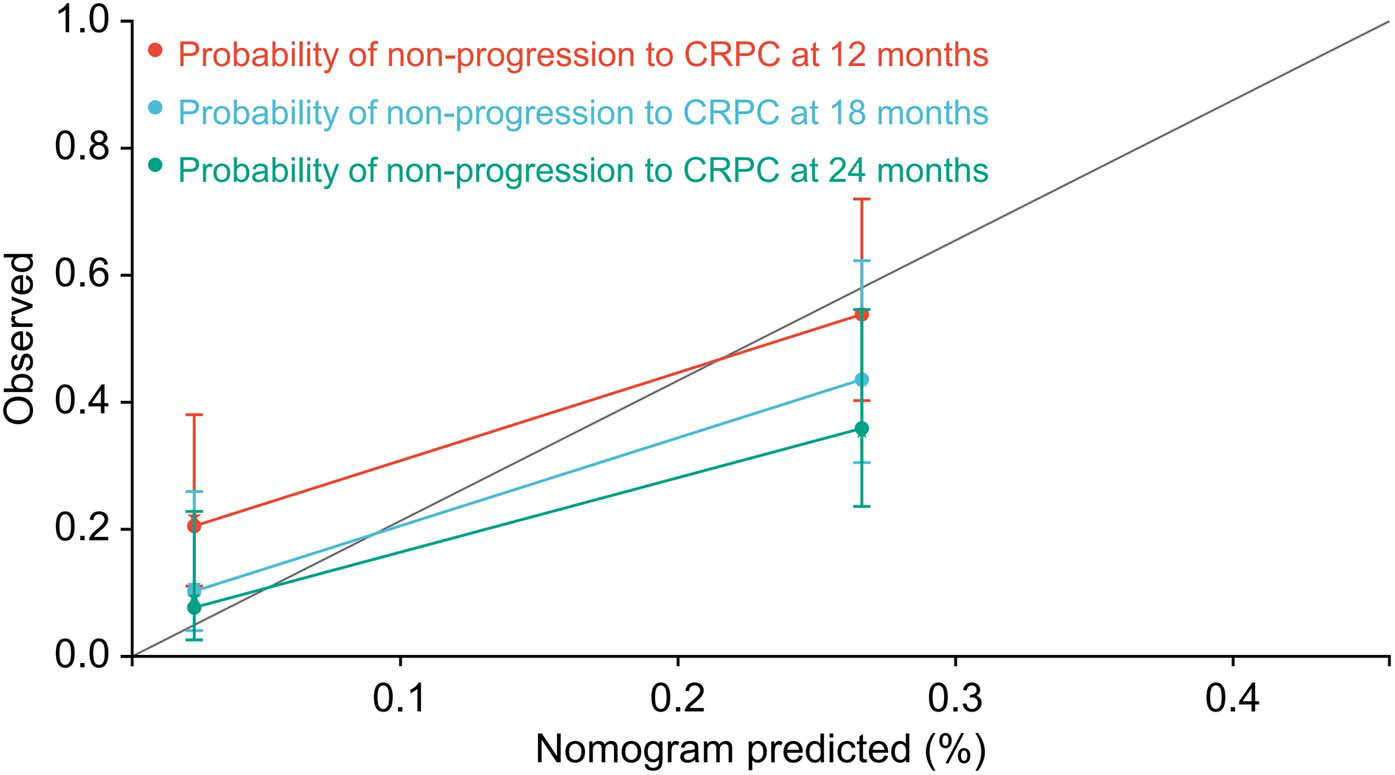

Supplement: Supplemental Figure S3 [file figs3.jpg]
